# Quantifying the Impoverishing Effects of Purchasing Medicines: A Cross-Country Comparison of the Affordability of Medicines in the Developing World

**DOI:** 10.1371/journal.pmed.1000333

**Published:** 2010-08-31

**Authors:** Laurens M. Niëns, Alexandra Cameron, Ellen Van de Poel, Margaret Ewen, Werner B. F. Brouwer, Richard Laing

**Affiliations:** 1 Institute for Medical Technology Assessment and Institute for Health Policy & Management, Erasmus University Rotterdam, The Netherlands; 2 Essential Medicines and Pharmaceutical Policies, World Health Organization, Geneva, Switzerland; 3 Health Action International Global, Amsterdam, The Netherlands; Harvard School of Public Health, United States of America

## Abstract

**Background:**

Increasing attention is being paid to the affordability of medicines in low- and middle-income countries (LICs and MICs) where medicines are often highly priced in relation to income levels. The impoverishing effect of medicine purchases can be estimated by determining pre- and postpayment incomes, which are then compared to a poverty line. Here we estimate the impoverishing effects of four medicines in 16 LICs and MICs using the impoverishment method as a metric of affordability.

**Methods and Findings:**

Affordability was assessed in terms of the proportion of the population being pushed below US$1.25 or US$2 per day poverty levels because of the purchase of medicines. The prices of salbutamol 100 mcg/dose inhaler, glibenclamide 5 mg cap/tab, atenolol 50 mg cap/tab, and amoxicillin 250 mg cap/tab were obtained from facility-based surveys undertaken using a standard measurement methodology. The World Bank's World Development Indicators provided household expenditure data and information on income distributions. In the countries studied, purchasing these medicines would impoverish large portions of the population (up to 86%). Originator brand products were less affordable than the lowest-priced generic equivalents. In the Philippines, for example, originator brand atenolol would push an additional 22% of the population below US$1.25 per day, whereas for the lowest priced generic equivalent this demographic shift is 7%. Given related prevalence figures, substantial numbers of people are affected by the unaffordability of medicines.

**Conclusions:**

Comparing medicine prices to available income in LICs and MICs shows that medicine purchases by individuals in those countries could lead to the impoverishment of large numbers of people. Action is needed to improve medicine affordability, such as promoting the use of quality assured, low-priced generics, and establishing health insurance systems.

*Please see later in the article for the Editors' Summary*

## Abstract 2

Laurens Niëns and colleagues estimate the impoverishing effects of four medicines in 16 low- and middle-income countries using the impoverishment method as a metric of affordability and show that medicine purchases could impoverish large numbers of people.

## Editors' Summary

**Background:**

In recent years, the international community has prioritized access to essential medicines, which has required focusing on the accessibility, availability, quality, and affordability of life-saving medicines and the development of appropriate data and research agendas to measure these components. Determining the degree of affordability of medicines, especially in low- and middle-income countries, is a complex process as the term affordability is vague. However, the cost of medicines is a major public health issue, especially as the majority of people in developing countries do not have health insurance and medicines freely provided through the public sector are often unavailable. Therefore, although countries have a legal obligation to make essential medicines available to those who need them at an affordable cost, poor people often have to pay for the medicines that they need when they are ill. Consequently, where medicine prices are high, people may have to forego treatment or they may go into debt if they decide to buy the necessary medicines.

**Why Was This Study Done?:**

The researchers wanted to show the impact of the cost of medicines on poorer populations by undertaking an analysis that quantified the proportion of people who would be pushed into poverty (an income level of US$1.25 or US$2 a day) because their only option is to pay out-of-pocket expenses for the life-saving medicines they need. The researchers referred to this consequence as the “impoverishing effect of a medicine.”

**What Did the Researchers Do and Find?:**

The researchers generated “impoverishment rates” of four medicines in 16 low- and middle-income countries by comparing households' daily per capita income before and after (the hypothetical) purchase of one of the following: a salbutamol 100 mcg/dose inhaler, glibenclamide 5 mg cap/tab, atenolol 50 mg cap/tab, and amoxicillin 250 mg cap/tab. This selection of drugs covers the treatment/management of three chronic diseases and one acute illness. The cost of each medicine was taken from standardized surveys, which report median patient prices for a selection of commonly used medicines in the private sector (the availability of essential medicines in the public sector is much lower so many people will depend on the private sector for their medicines) for both originator brand and lowest priced generic products. If the prepayment income was above the US$1.25 (or US$2) poverty line and the postpayment income fell below these lines, purchasing these medicines at current prices impoverishes people.

According to the results of this analysis, a substantial proportion (up to 86%) of the population in the countries studied would be pushed into poverty as a result of purchasing one of the four selected medicines. Furthermore, the lowest priced generic versions of each medicine were generally substantially more affordable than originator brand products. For example, in the Philippines, purchasing originator brand atenolol would push an additional 22% of the population below US$1.25 per day compared to 7% if the lowest priced generic equivalent was bought instead. In effect, purchasing essential medicines for both chronic and acute conditions could impoverish large numbers of people, especially if originator brand products are bought.

**What Do These Findings Mean?:**

Although the purchasing of medicines represents only part of the costs associated with the management of an illness, it is clear that the high cost of medicines have catastrophic effects on poor people. In addition, as the treatment of chronic conditions often requires a combination of medicines, the cost of treating and managing a chronic condition such as asthma, diabetes, and cardiovascular disease is likely to be even more unaffordable than what is reported in this study. Therefore concerted action is urgently required to improve medicine affordability and prevent poor populations from being pushed further into poverty. Such action could include: governments, civil society organizations, and others making access to essential medicines more of a priority and to consider this strategy as an integral part of reducing poverty; the development, implementation, and enforcement of sound national and international price policies; actively promoting the use of quality assured, low-cost generic drugs; ensuring the availability of essential medicines in the public sector at little or no charge to poor people; establishing health insurance systems with outpatient medicine benefits; encouraging pharmaceutical companies to differentially price medicines that are still subject to patent restrictions.

**Additional Information:**

Please access these Web sites via the online version of this summary at http://dx.doi.org/10.1371/journal.pmed.1000333.

- For a comprehensive resource for medicine prices, availability, and affordability, see Health Action International
- Guidelines about access to essential medicines and pharmaceutical policies can be found at WHO
- Transparency Alliance provides more information about medicines
- Access to essential medicines has become a key campaign topic; for more information see Médecins Sans Frontières (Doctors without Borders)

## Introduction

In developing countries the cost of medicines accounts for a relatively large portion of total healthcare costs [1]–[4]. As the majority of people in developing countries do not have health insurance [5] and medicines provided free through the public sector are often unavailable [4], medicines are often paid for out of pocket at the time of illness. Consequently, where medicine prices are high, people may be unable to procure them and therefore forego treatment or they may go into debt. For this reason, the World Health Organization (WHO) has designated affordable prices as a determinant of access to medicines (together with rational selection and use, sustainable financing, and reliable health and supply systems) [6]. In several international treaties, access to healthcare has been established as a right [7],[8]. States have a legal obligation to make essential medicines available to those who need them at an affordable cost. Determining the degree of affordability of medicines, especially in low- and middle-income countries (LICs and MICs), is an important, yet complex undertaking as affordability is a vague concept.

Medicine affordability has been investigated in terms of the days' wages that a country's lowest paid unskilled government worker (LPGW) needs to spend on a standard course of treatment [4],[9]. However, this metric is limited because it does not provide insight into the affordability of medicines for the often large sections of the population that earn less than the LPGW [4],[10]. Recently, Niëns et al. have proposed two alternative methods to gain insight into the affordability of medicines in the developing world [11]. A first method focuses on the catastrophic impact of expenditures on medicines, while the second approach consists of studying the impoverishing effect of these expenditures. Here we discuss the application of the latter approach and present the results of a cross-country analysis of the affordability of four medicines in 16 developing countries.

## Methods

Our measurement of the affordability of medicines is based on the approach taken by Van Doorslaer et al. [3], who reassessed poverty estimates in 11 Asian countries after taking into account household expenditures on health care. The impoverishment approach has also been used in other fields of study such as housing affordability [12],[13] and health insurance [14].

The impoverishing effect of a medicine is defined in terms of the percentage of the population that would be pushed below an income level of US$1.25 or US$2 per day when having to purchase the medicine. Although different income levels have been used/proposed [3],[15], the US$1.25 and US$2 poverty lines were chosen because they are the most recent widely recognized poverty indicators as used by the World Bank [16]. Thus, the approach essentially compares households' daily per capita income before and after (the hypothetical) procurement of a medicine. If the prepayment income is above the US$1.25 (or US$2) poverty line and the postpayment income falls below these lines, purchasing the medicine impoverishes people. We used this method to generate “impoverishment rates,” which denote the percentage of the population that would become impoverished. The unaffordability of a medicine then refers to the percentage of the population that either already is or would fall below the poverty line when having to procure the medicine. First we consider the affordability of medicines in the total population at risk of becoming ill. We also indicate, using prevalence rates for the three chronic diseases, the expected number of patients actually affected.

### Data

To conduct the first analysis, three types of data were required per country: medicine prices, aggregated income data, and information on the income distribution. In calculating expected numbers of patients affected, prevalence data are also required. Medicine prices were taken from standardized surveys using the WHO/Health Action International (HAI) price measurement methodology, which report median patient prices for a selection of commonly used medicines in the private sector, for both originator brand (OB) and lowest priced generic (LPG) products [17]. We focused on the private sector because the availability of essential medicines in the public sector is much lower [4]. In the countries studied here, therefore, many people will depend on the private sector for their medicines.

The World Bank's World Development Indicators (WDIs) provided Household Final Consumption Expenditure (HHFCE) data and information on income distribution [18]. Although WDIs have shortcomings (highlighted in the Discussion section), they have the advantage of being available for a wide range of countries. Moreover, in this context commonly used household surveys are often not available on a yearly basis and are not conducted in a standardized way, limiting the comparability of results across countries and over time [3],[19]. Here we use an affordability measure that can be quite easily applied in LICs and MICs where the use of more detailed household survey data may be limited.

HHFCE was selected as an aggregate income measure rather than gross domestic product (GDP) per capita as it better reflects households' resources [19], while GDP also includes consumption, gross investment, and net trade. Because the WDI did not provide any information on HHFCE for Nigeria and Yemen, the Economist Intelligence Unit (EIU) nominal private consumption figure was used for these countries [20]. For simplicity, we refer to “income” as measured by HHFCE or nominal private consumption. Apart from average income, the WDIs also provide some information on a country's income distribution by listing the proportion of total income earned in seven income groups; five income quintiles, with the poorest and richest quintiles split into deciles.

At the time of analysis, medicine price surveys were available for 53 countries. In large countries such as India and China, price surveys were carried out on a state or provincial level [4]. Because the WDIs do not provide state-level income distributions, HHFCE, and population figures, these countries were excluded from the current study. To ensure cross-country comparability, the analysis was limited to countries where income distributions (WDI data) were available from the year 2000 onwards. We used WDI income data from the same year as the WHO/HAI price data. Data on income distributions for the same year were used when possible, if not, the most recent income distribution data prior to the year of the price and income data were used.

Table 1 provides an overview of all countries and data used in this study. When discussing results, countries were grouped into LICs and MICs according to the 2008 World Bank's classification [21]. Sixteen countries were selected on the basis of the availability of WHO/HAI data. They are not representative of the developing world as a whole. However, as these countries vary substantially in terms of economic development, health care infrastructure, and medicine prices, they provide an interesting sample to study affordability of medicines.

**Table 1:** Overview of countries studied and years of data sources used.

Countries	Medicine Price Survey and WDI Income Data	WDI Data on Income Distribution
***LIC***		
Kyrgyzstan	2005	2003
Mali	2004	2001
Nigeria	2004^a^	2003
Pakistan	2004	2002
Tajikistan	2005	2004
Tanzania	2003	2000
Uganda	2004	2002
Uzbekistan	2004	2003
Yemen	2006^a^	2005
***MIC***		
El Salvador	2006	2002
Indonesia	2004	2002
Jordan	2004	2002
Mongolia	2004	2002
Peru	2005	2003
Philippines	2005	2003
Tunisia	2004	2000

a Nominal private consumption from EIU was used.

We selected four medicines for which price data were available for the majority of countries and for which treatment regimens are relatively standard across countries. While these may not lead to results that are in a strict sense generalisable, they provide valuable insight into the affordability of common medicines in the selected countries. Table 2 lists the medicine, the ill health conditions for which these medicines are used, the total number of units per treatment course, and the treatment duration in days [17]. Three of the four study medicines are used to treat chronic conditions (asthma, diabetes, and hypertension). For each of these, we also calculated the expected numbers of patients becoming impoverished, using the prevalence data shown in Table S1. We could not do this for adult respiratory infection because of unavailability of comparable prevalence data.

**Table 2:** Description of studied medicines.

Medicine Name	Ill Health Condition	Medicine Strength per Dose	Total *n* of Doses per Treatment	Dosage Form	Treatment Duration (d)
Salbutamol inhaler	Asthma	100 mcg	200	Inhaler	30 (1 inhaler)
Glibenclamide	Diabetes	5 mg	60	Capsule/tablet	30
Atenolol	Hypertension	50 mg	30	Capsule/tablet	30
Amoxicillin	Adult Respiratory Infection	250 mg	21	Capsule/tablet	7

The emphasis on medicines for chronic disease is justified by the fact that these conditions require ongoing, usually lifelong expenditures, making it more difficult for households to use financing strategies like borrowing and selling assets [22]. Table 2 shows that the treatment duration for these medicines was set at 30 d to represent the monthly treatment costs. The affordability of one acute condition (adult respiratory infection) treated with a 7-d treatment course of amoxicillin was also studied. Recently, the WHO increased the guidelines for treatment of adult respiratory infection with amoxicillin to a daily regimen of three times 500 mg amoxicillin. This change implies that the affordability of this medicine is likely to be lower than reported here [23].

### Calculation Methods

Our method of estimating the impoverishing effect of procuring medicines was based on the method used by Van Doorslaer et al. [3]. However, using aggregate data requires some simplifying assumptions about the income distribution across population groups. For a detailed discussion of the method used to calculate the impoverishing effect of medicines, we refer to Niëns et al. [11]. The basic idea is to compare poverty estimates before and after a (potential) purchase of the medicines listed in Table 1. Average per capita income within each income group is estimated by combining information on the proportion of total income earned across income groups with data on the HHFCE (as provided by the WDIs). As only data on average income in the different quintiles and deciles were available, we assumed linearity of the income distribution within these relevant groups in which the US$1.25 and US$2 poverty lines were located in calculating poverty and impoverishment. The proportion of the population that would earn less than US$1.25 or US$2 per day after buying a medicine but not before would therefore be impoverished because of purchasing medicines. The medicine is deemed affordable for the proportion of the population that would remain above the poverty line after having purchased it. We also estimated the actual number of patients with one of the three chronic illnesses for which the medicine is unaffordable. To make this estimation, we used prevalence rates from various data sources and again assume that the respective disease is evenly spread over the income distribution.

Because HHFCE is measured in current US$, we recalculated the US$1.25 and US$2 poverty lines to US$ values for the HAI/WHO survey year. HAI/WHO medicine prices were expressed in US$ for the same year.

## Results

Table 3 presents the percentages of the population that are pushed below the poverty line owing to the purchasing of each of the four study medicines, both LPG and OB products.

**Table 3:** Percentage of the population below the poverty line before procurement of the medicines and the population drawn below the poverty line by expenditures on these medicines.

Percent of Population below Poverty Lines and LPGW Wage, before Medicine Purchase					Percent of Population below Poverty Line after Medicine Purchase															
					Salbutamol Inhaler				Glibenclamide				Atenolol				Amoxicillin			
Country	GDP/Cap (Current US$)^b^	US$1.25	US$2	LPGW wage	US$1.25		US$2		US$1.25		US$2		US$1.25		US$2		US$1.25		US$2	
					OB	LPG	OB	LPG	OB	LPG	OB	LPG	OB	LPG	OB	LPG	OB	LPG	OB	LPG
***LIC***																				
Kyrgyzstan	478	0	14	10	8	5	22	18	a	2	a	2	a	1	a	15	a	12	a	26
Mali	422	37	61	85	51	50	67	69	66	53	76	65	a	a	a	a	a	a	a	a
Nigeria	639	56	77	90	71	a	77	a	71	71	79	79	67	63	83	81	79	68	80	84
Pakistan	644	—	8	46	0	a	13	a	0	0	13	12	1	0	17	12	4	4	21	21
Tajikistan	354	10	32	1	a	19	a	40	a	11	a	33	a	12	a	34	a	28	a	50
Tanzania	286	50	77	96	a	60	a	82	a	58	a	81	a	57	a	80	a	61	a	82
Uganda	305	52	73	90	59	54	77	74	a	53	a	74	72	53	85	74	74	54	86	74
Uzbekistan	465	17	38	68	28	24	50	46	a	22	a	43	a	19	a	41	a	35	a	58
Yemen	882	7	22	87	14	10	31	26	29	10	47	26	20	9	38	25	a	12	a	28
***MIC***																				
El Salvador	3,067	7	11	59	9	9	14	14	17	11	21	16	15	12	19	17	21	a	26	a
Indonesia	1,187	—	4	71	11	a	20	a	13	0	36	6	15	0	38	12	20	0	43	9
Jordan	2,157	—	1	58	0	0	4	2	0	0	6	3	1	0	8	4	19	3	26	10
Mongolia	721	2	13	81	a	7	a	20	a	6	a	19	a	4	a	19	a	8	a	13
Peru	2,852	2	9	64	7	5	14	11	a	5	a	11	13	4	20	10	27	7	33	14
Philippines	1,156	5	21	88	16	12	32	28	23	13	38	29	27	12	41	28	23	17	37	32
Tunisia	2,832	—	1	73	0	a	2	a	0	0	5	2	a	0	a	3	a	a	a	a

The sum of both gives the proportion of the population for which the medicine is unaffordable.

a No reliable medicine price estimate was possible due to lack of data.

b Source: WDI [18].

For each country, Table 3 first highlights the proportion of the population already below the US$1.25 and US$2 poverty lines without purchasing these medicines. These poverty estimates correlate highly with the commonly used (household-survey based) estimates from the United Nations Development Program with Pearson correlation coefficients [24] equal to 0.90 for the proportion of the population below the US$1.25 poverty line, and 0.86 for the proportion below the US$2 poverty line. Table 3 also shows the proportion of the population earning less than the LPGW, which varies widely across countries; from only 1% in Tajikistan to 96% in Tanzania. This cross-country variability represents one of the limitations of the LPGW metric as used by the WHO/HAI methodology [9].

Comparing the proportion of the population below the US$1.25 and US$2 poverty lines before and after procurement of medicines gives insight into the impoverishing effect of medicine procurement. By adding the proportion of the population already living below the US$1.25 and US$2 poverty lines to the group that would fall below these poverty lines when procuring the medicines, we get the proportion of the population for which the four medicines are unaffordable.

The results in Table 3 illustrate that the impoverishing effect of medicines varies substantially between OB and LPG products. For example in Yemen, a LIC where 7% of the population lives on a prepayment income of less than US$1.25 a day, OB glibenclamide purchased in the private sector would impoverish an additional 22% of the population versus 3% for the LPG equivalent. In Nigeria, a LIC where 56% of the population lives below US$1.25 per day, purchasing amoxicillin from the private sector would impoverish an additional 23% if the OB is bought and 12% if buying the LPG.

Rather than showing proportions of the population, Table 4 presents both the absolute number of individuals that would be pushed into poverty owing to the cost of buying medicines from the private sector (“Impoverished” column) and the number of people for which medicines are unaffordable (“Unaffordable” column). Besides absolute figures, in Table 5 we present the relative change of the poverty estimates for the total population studied as well as for the patient population. So, if 40% of the population is initially above the poverty line, while only 30% would remain above after purchasing medicines, this proportion is 25% (10% out of 40% are impoverished). These numbers are listed for all four medicines, both OB and LPG. The total population of the 16 countries analyzed amounts to over 775 million people, of which approximately 126 million live on less than US$1.25 and 209 million on less than US$2 per day, respectively. Table 4 illustrates that across this set of 16 developing countries, for respectively almost one-fourth and two-fifths of the total population, essential medicines are unaffordable using the US$1.25 and US$2 poverty line.

**Table 4:** Absolute impoverishment and unaffordability estimates of medicines procured in the private sector for the total and chronic patient populations across 16 countries (rounded to millions).

	Medicine	Under US$1.25				Under US$2			
		OB		LPG		OB		LPG	
		Impoverished	Unaffordable	Impoverished	Unaffordable	Impoverished	Unaffordable	Impoverished	Unaffordable
**Total Population**	Salbutamol inhaler	64	190	16	142	71	280	23	233
	Glibenclamide	72	198	36	162	112	321	35	244
	Atenolol	80	206	20	146	133	343	50	259
	Amoxicillin	111	237	46	172	144	354	72	281
**Chronic Patient Population**	Salbutamol inhaler	3	9	1	7	2	13	1	12
	Glibenclamide	3	8	2	6	5	14	2	11
	Atenolol	19	58	6	44	29	92	10	73

Impoverished, the number of people that would be pushed below the US$1.25 and US$2 poverty lines if the total population had to buy the respective medicine.

Unaffordable, the total number of people for which the respective medicine can be considered unaffordable.

**Table 5:** The relative change of the poverty estimates (i.e., the impoverished population expressed as a proportion of the population initially above the poverty line).

	Medicine	Additional Percentage under US$1.25		Additional Percentage under US$2	
		OB	LPG	OB	LPG
**Total Population**	Salbutamol inhaler	10	2	13	4
	Glibenclamide	11	6	20	6
	Atenolol	12	3	23	9
	Amoxicillin	17	7	25	13
**Chronic Patient Population**	Salbutamol inhaler	10	2	10	3
	Glibenclamide	10	5	18	5
	Atenolol	12	4	21	7

The upper half of Table 4 shows the proportions of the total population for which medicines would be unaffordable when having to procure them. The actual number of people affected by this unaffordability (in terms of experiencing the disease) depends on the prevalence of diseases as well. Therefore, the lower half of Table 4 also shows the expected absolute number of patients affected by the unaffordability of medicines using the prevalence rates listed in Table S1. As the prevalence rates of hypertension are substantially higher than those of asthma and diabetes, the impoverishing effect, and therefore also the unaffordability, of atenolol is substantially higher than that for the other medicines. In this approach, given the height and distribution of income, impoverishment is determined by both medicine prices and prevalence rates for the relevant diseases.

## Discussion

The results illustrate that substantial proportions of the population would be pushed into poverty as a result of medicine procurement, implying that in many countries affordability of these treatments is low. In the private sector, LPGs were generally substantially more affordable than OB products. Thus, increasing the use of quality-assured generics could reduce the impoverishing effect of medicines. This use of generics, in turn, could bring about improvements in the health status of these populations by avoiding low compliance to recommended dosages or duration of treatment, resulting in problems such as sustained hypertension, elevated blood glucose levels, or the promotion of bacterial resistance due to too short courses of antibiotics.

Our calculation method has the advantage of allowing for comparisons of medicine-induced impoverishment across time and across countries using widely available aggregate data. The method, therefore, is useful and generalisable for studying the affordability of a wide range of goods and health care services. The use of such data also brings some limitations, which are discussed in further detail in Niëns et al. [11]. First, dividing HHFCE by total population to get an estimate of income per capita assumes that each household is the same size. However, poor households are generally larger than their richer counterparts [25]. This discrepancy causes the average income per capita to be overestimated in the lower income groups, making our affordability estimates rather conservative. Second, the assumption of linearity of the income distribution between income groups is also likely to lead to an overestimation of average incomes across the income distribution and therefore to a downward bias in our results. We also assumed a linear distribution of illness over the income distribution to calculate expected numbers of affected people. Although, in general, disease may be more prevalent in low income groups, which would imply conservative estimates of unaffordability, this also depends on the exact diseases studied. Moreover, it is clear that considering only medicine costs, for four medicines independently, merely demonstrates the larger problem of medicine and health care affordability. The treatment of chronic conditions often requires a combination of medicines and is therefore likely to be even more unaffordable than what is reported here [4]. For chronic asthma patients, for example, appropriate management of their disease requires use of both salbutamol and beclometasone inhalers for treatment and prevention [17]. Due to the lack of available price information on beclometasone inhalers (because of poor availability), it was not possible to include this medicine in the analysis. As such, the true affordability of asthma treatment is likely to be lower than reported in Tables 3 and 4. Having said this, the medicines studied in this paper are commonly used to treat ill health conditions from which considerable proportions of the population in the developing world suffer, as is also illustrated in Table S1 [26]. As such, low affordability of these medicines is likely to signal a more general problem of low affordability of medicines in LIC and MIC. Further, it should be noted that comparability of impoverishment rates for acute and chronic conditions may be limited. If people suffer from an adult respiratory infection, on average three times per year, and are able to shift resources over time, the impoverishment rates for amoxicillin should be interpreted with caution. Further research is needed on this issue, for example by calculating affordability for standardized time periods taking into account the relevant incidence rates of respiratory infections.

Notwithstanding these limitations, this study provides useful insights into the affordability of these four medicines in the developing world. When medicine prices are known, the methods used, as they rely on easily obtainable aggregated data, can be used to compare affordability of medicines across countries and over time. Clearly, medicines represent only a part of the costs associated with the management of an illness. Other costs, such as for diagnostics, physician consultations, transport costs to clinics, lost work time, etc., place an additional burden on household finances in developing countries. However, given the relatively large share of health care costs for medicines in developing countries [1]–[4], medicine affordability is likely to be an important determinant to access to treatment.

This study shows high medicine costs can push large groups of patients into poverty. These results call for action, both by governments, civil society organizations, and others, to make access to essential medicines a priority, and not only to ensure access to necessary medicines, but also as a component in the context of reducing poverty. Possible lines of action include developing, implementing, and enforcing sound national and international price policies. In the short term these policies could encompass, for example, restrictions on supply chain mark-ups, tax exemptions, and regulating prices for end-users. Promoting the use of quality-assured, low-cost generics, for example, through preferential registration procedures, is also an important strategy [4]. In the public sector, ensuring availability of essential medicines at little or no charge to the poor is critical. In the longer term, establishing health insurance systems with outpatient medicine benefits seems crucial to avoid poverty due to health shocks (and poor health due to poverty). Innovative approaches, such as using private distribution systems to supply subsidized medicines to chronic disease patients, should also be considered. For medicines that are still subject to patent restrictions, pharmaceutical companies should be encouraged to differentially price these products, as is the case with antiretrovirals [27]. Countries also have the option of using compulsory licensing to oblige patent holders to grant its use to the state or others [28], as was recently done by Thailand [29],[30].

When resources are limited, those in greatest need, such as people suffering from chronic disease who earn less than US$1.25 per day, should benefit from state and/or donor actions. The price in terms of health losses due to unaffordable medicines is something we cannot afford.

## Supporting Information

Alternative Language Abstract S1 Abstract translated into French by Ellen Van de Poel and Gabriela Flores.
(0.02 MB DOC)

Alternative Language Abstract S2 Abstract translated into Spanish by Laurens M. Niëns and Isaac Corro Ramos.
(0.02 MB DOC)

Table S1 The prevalence of three chronic diseases.
(0.05 MB DOC)

## Abbreviations

EIU: Economist Intelligence Unit
GDP: gross domestic product
HAI: Health Action International
HHFCE: household final consumption expenditure
LIC: low-income country
LPG: lowest priced generic
LPGW: lowest paid government worker
MIC: middle-income country
OB: originator brand
WDI: world development indicator
WHO: World Health Organization
