# Supplementary material for: Quantifying the Impoverishing Effects of Purchasing Medicines: A Cross-Country Comparison of the Affordability of Medicines in the Developing World
Source: PLoS Med. 2010 Aug 31;7(8):e1000333. doi: 10.1371/journal.pmed.1000333 (PMC2930876; doi:10.1371/journal.pmed.1000333)
Supplement: Alternative Language Abstract S1 — Abstract translated into French by Ellen Van de Poel and Gabriela Flores. (0.02 MB DOC) [file pmed.1000333.s001.doc]

**Quantifier les effets d'appauvrissement d'achat de médicaments
Une comparaison entre les pays sur l'accessibilité financière des médicaments dans le monde en développement**

Laurens M. Niëns^1*^, Alexandra Cameron^2^, Ellen Van de Poel^1^, Margaret Ewen^3^, Werner B.F. Brouwer^1^, Richard Laing^2^.

^1^ Institute for Medical Technology Assessment and institute for Health Policy & Management, Erasmus University Rotterdam, Netherlands.

^2^ Essential Medicines and Pharmaceutical Policies, World Health Organization, Geneva, Switzerland.

^3^ Health Action International Global, Amsterdam, Netherlands.

**Résumé**CONTEXTE

L'accessibilité de médicaments dans les pays à faibles et moyens revenus, où les médicaments sont souvent très coûteux par rapport aux niveaux des revenus, est sous une attention croissante. L’effet des dépenses en médicaments sur la pauvreté peut être estimé en déterminant le revenu avant et après l’achat des médicaments, qui sera comparé avec le seuil de pauvreté. Dans cet article, nous estimons les effets d'appauvrissement de quatre médicaments dans seize pays à revenus faibles et moyens pour mesurer l’accessibilité financière des médicaments.

MÉTHODES ET RÉSULTATS

L’accessibilité financière des médicaments est évaluée en termes de la proportion de la population poussée sous le seuil de pauvreté de USD1.25 ou de USD2 par jour par achat de médicaments. Les prix de salbutamol inhalateur 100mcg/dosis, glibenclamide 5 mg/capsule, aténolol 50 mg/capsule et amoxicilline 250 mg/capsule ont été obtenus à partir d’enquêtes auprès des établissements de santé selon les méthodes standard d’enquêtes. Les indicateurs de développement Humain de la Banque mondiale nous ont fourni des données sur les dépenses des ménages et la répartition des revenus.
Dans les pays étudiés, l'achat de ces médicaments appauvri une grande partie de la population (jusqu'à 84%). En outre, les produits de marque se sont révélés moins accessibles que les équivalents génériques. Aux Philippines, par exemple, la marque atenelol pousse 22% de la population en dessous du seuil de USD1.25 par jour tandis que pour l'équivalent générique, ayant le prix le plus bas, le taux d’appauvrissement est de 7%.

CONCLUSIONS

Une comparaison des prix des médicaments et des revenus disponibles dans les pays à faibles et moyens revenus montre que l'achat de médicaments pourrait appauvrir beaucoup de gens. Afin d'améliorer l'accessibilité aux médicaments, il est nécessaire de prendre des mesures telles que la promotion de l'utilisation des médicaments génériques de qualité, ayant des prix bas et la prise en charges des médicaments par une assurance maladie.

* Correspondence: Laurens Niëns, institute of Health Policy and Management, Erasmus University Rotterdam, PO Box 1738, 3000 DR Rotterdam, Netherlands. E-mail: niens@bmg.eur.nl

Translation by Dr. Ellen Van de Poel (co-author of this paper) and Dr. Gabriela Flores, University of Lausanne, Switzerland, formerly of the Erasmus University, Rotterdam.
