# Supplementary material for: Quantifying the Impoverishing Effects of Purchasing Medicines: A Cross-Country Comparison of the Affordability of Medicines in the Developing World
Source: PLoS Med. 2010 Aug 31;7(8):e1000333. doi: 10.1371/journal.pmed.1000333 (PMC2930876; doi:10.1371/journal.pmed.1000333)
Supplement: Alternative Language Abstract S2 — Abstract translated into Spanish by Laurens M. Niëns and Isaac Corro Ramos. (0.02 MB DOC) [file pmed.1000333.s002.doc]

**Cuantificación de los Efectos Empobrecedores en la Compra de Medicamentos
Una comparación entre países sobre la asequibilidad de medicamentos en el mundo en desarrollo**
Laurens M. Niëns^1*^, Alexandra Cameron^2^, Ellen Van de Poel^1^, Margaret Ewen^3^, Werner B.F. Brouwer^1^, Richard Laing^2^.

^1^ Institute for Medical Technology Assessment and institute for Health Policy & Management, Erasmus University Rotterdam, Netherlands.

^2^ Essential Medicines and Pharmaceutical Policies, World Health Organization, Geneva, Switzerland.

^3^ Health Action International Global, Amsterdam, Netherlands.

**Resumen**

CONTEXTO **La asequibilidad de medicamentos en países de ingresos bajos y medios, donde los medicamentos son a menudo muy caros en relación con los niveles de ingresos, está siendo objeto de una creciente atención. El efecto de la pobreza en las compras de medicamentos puede ser estimado determinando los ingresos anteriores y posteriores al pago de los medicamentos, los cuales serán comparados con el umbral de pobreza. En este trabajo se estiman los efectos empobrecedores de cuatro medicamentos en dieciséis países de ingresos bajos y medios usando el método de empobrecimiento como medida de asequibilidad.**

MÉTODOS Y RESULTADOS **La asequibilidad ha sido evaluada en términos de la proporción de la población que, debido a la compra de medicamentos, se encuentra por debajo de los niveles de pobreza de USD1.25 o USD2 por día. Los precios del inhalador salbutamol 100mcg/dosis, de la glibenclamida 5mg capsula/tableta, del atenolol 50 mg capsula/tableta y de la amoxicilina 250 mg capsula/tableta, han sido obtenidos mediante encuestas realizadas en los establecimientos y usando una metodología de medición estándar. Los Indicadores de Desarrollo Mundial del Banco Mundial nos han proporcionado datos acerca del gasto en los hogares y la distribución de los ingresos.**

**En los países estudiados, la compra de estos medicamentos empobrecería a una gran parte de la población (hasta un 84%). Además, los productos de marca registrada han resultad son menos asequibles que los equivalentes genéricos. En Filipinas, por ejemplo, el atenelol de marca provocaría que otro 22% de la población estuviera por debajo de USD1.25 por día mientras que para el equivalente genérico de más bajo precio esto se reduciría a un 7%. En la práctica, teniendo en cuenta las cifras relacionadas con la prevalencia, un número considerable de personas se ven afectadas por el elevado precio de los medicamentos.**

CONCLUSIONES **La comparación entre los precios de medicamentos y los ingresos disponibles en países de ingresos bajos y medios nos muestra que la compra de medicamento podría empobrecer a un gran número de personas. Para mejorar la asequibilidad en la medicina, es necesario adoptar medidas tales como la promoción del uso de medicamentos genéricos de bajo precio y calidad garantizada o la implantación de un seguro médico.**

* Correspondencia a: Laurens Niëns, institute of Health Policy and Management, Erasmus University Rotterdam, PO Box 1738, 3000 DR Rotterdam, Netherlands. E-mail: niens@bmg.eur.nl

Translated by the corresponding author, Laurens Niens, and Dr. Isaac Corro Ramos, Dr. Niens’ colleague from the Erasmus University, Rotterdam.
