# Supplementary material for: Quantifying the Impoverishing Effects of Purchasing Medicines: A Cross-Country Comparison of the Affordability of Medicines in the Developing World
Source: PLoS Med. 2010 Aug 31;7(8):e1000333. doi: 10.1371/journal.pmed.1000333 (PMC2930876; doi:10.1371/journal.pmed.1000333)
Supplement: Table S1 — The prevalence of three chronic diseases. (0.05 MB DOC) [file pmed.1000333.s003.doc]

|  | **Proportion of the population suffering from** | | |
| --- | --- | --- | --- |
| **Condition** | **asthma** | **diabetes** | **hypertension** |
| ***Low-income*** |  |  |  |
| Kyrgyzstan | 4,3 | 4,3 | 28,0 |
| Mali | 2,6 | 3,3 | 27,5 |
| Nigeria | 5,7 | 3,9 | 34,8 |
| Pakistan | 4,1 | 7,6 | 24,0 |
| Tajikistan | 4,3 | 1,0 | 24,0 |
| Tanzania | 4,4 | 2,6 | 27,5 |
| Uganda | 4,4 | 1,7 | 27,5 |
| Uzbekistan | 4,3 | 7,0 | 7,6 |
| Yemen | 5,8 | 2,5 | 9,7 |
| ***Middle- income*** |  |  |  |
| El Salvador | 3,8 | 7,8 | 42,0 |
| Indonesia | 3,3 | 4,6 | 23,3 |
| Jordan | 5,8 | 7,5 | 22,2 |
| Mongolia | 2,1 | 1,3 | 42,0 |
| Peru | 9,9 | 5,6 | 15,2 |
| Philippines | 7,9 | 6,7 | 20,2 |
| Tunisia | 3,7 | 8,5 | 33,0 |

**Table S1: the prevalence of three chronic diseases.**

Notes:

Diabetes prevalence data was retrieved from the international diabetes federation’s data website (<http://www.diabetesatlas.org/>).

Asthma prevalence comes from the Global Initiative for Asthma’s Burden of Asthma Report (<http://www.ginasthma.com/ReportItem.asp?l1=2&l2=2&intId=94>).

Hypertension prevalence comes from WHO infobase online (<https://apps.who.int/infobase/report.aspx>) and a Lancet article by Kearney et al 2005.
